# Knowledge, attitudes, and practices of Australian allied hearing-healthcare professionals: survey on comorbid hearing loss and cognitive impairment

**DOI:** 10.3389/fmed.2024.1412475

**Published:** 2024-08-23

**Authors:** Dona M. P. Jayakody, Eunkyeong Grace Je, Isabelle Livings, Paul McIlhiney, Michelle Trevenen, Damir Kekez, Nahal Mavaddat

**Affiliations:** ^1^Ear Science Institute Australia, Subiaco, WA, Australia; ^2^Centre for Ear Sciences, Medical School, The University of Western Australia, Crawley, WA, Australia; ^3^WA Centre for Health and Ageing, Medical School, The University of Western Australia, Crawley, WA, Australia; ^4^School of Allied Health, Curtin University, Bentley, WA, Australia; ^5^School of Human Sciences, The University of Western Australia, Perth, WA, Australia; ^6^Medical School, The University of Western Australia, Crawley, WA, Australia; ^7^School of Physics, Mathematics and Computing, University of Western Australia, Perth, WA, Australia

**Keywords:** dementia, audiology, KAP survey, quality of care, health service delivery

## Abstract

**Purpose:**

As hearing loss is a modifiable risk factor of dementia, allied hearing-healthcare professionals (AHHPs) frequently see older patients who are affected by both conditions. However, little is known about how well Australian AHHP’s understand the complexities of providing care to patients with comorbid hearing loss and dementia, as well as their associated views and practices. Thus, the current study used a survey to explore the knowledge, attitudes, and practices (KAPs) of Australian AHHPs in managing comorbid patients.

**Materials and methods:**

A cross-sectional design was used, wherein a KAP survey was developed and distributed to eligible AHHPs via Qualtrics. Data were analysed with descriptive statistics and binary logistic regression.

**Results:**

101 Australian AHHPs met inclusion criteria (2.5% of approximately 4,000 invited AHHPs), and participated in the study. Although participants generally possessed a high level of knowledge for the association between hearing loss and cognitive impairment, their specific knowledge and practices in relation to cognitive screening tests and referral pathways was limited. Participants also expressed mostly positive attitudes towards their role in assisting patients with comorbid hearing loss and dementia. Furthermore, our results suggested that some KAPs relevant to comorbid patients differed based on sex, qualification, and ethnicity.

**Conclusion:**

This study identified gaps in the knowledge and practices of Australian AHHPs with regard to the complexities of addressing comorbid cognitive impairment and hearing loss. These findings will help to develop training programs to empower AHHPs to deliver optimal healthcare services to comorbid patients.

## 1 Introduction

Dementia is a progressive disorder characterised by cognitive impairments that severely affect independence and activities of daily living mostly in those aged 60 years and over (1), and was estimated to affect up to 472,000 Australians in 2021 (2). Mild Cognitive Impairment (MCI), meanwhile, is characterised by cognitive impairment(s)—namely of memory and/or executive functions—that are debilitating but not sufficiently detrimental to an individual’s independence (3); it is generally considered a prodromal state for dementia. MCI has received increasing scientific interest due to the potential for early intervention and the lack of effective treatments for more advanced dementia (4). Furthermore, it is estimated that up to 40% of MCI and dementia cases could be prevented or delayed by addressing associated modifiable risk factors. Of these factors, hearing loss has the highest population attributable risk factor of 8.2% (5).

Indeed, hearing loss is itself a major chronic illness, significantly affecting an estimated 403.3 million people globally in 2019 (6). Of those affected, 62.1% are aged 50 years or over, with sharp increases in prevalence after the age of 60 (6). Furthermore, untreated hearing loss is associated with emotional loneliness (7), social isolation (8) and depression (9). Several studies have also found hearing loss to be associated with cognitive impairment (10–12) and dementia (13–16), with hearing loss of mild, moderate, and severe degrees increasing dementia rates by two, three, and five times, respectively (13). Numerous studies also show that hearing intervention, either with hearing aids (17, 18) or cochlear implants (19–21), decelerates cognitive deterioration. However, findings from randomised-control studies such as the ACHIEVE (22) and HearCog (23) are awaited to provide further insight into whether hearing intervention prevents, or reduces, the rate of cognitive decline in hearing-impaired older adults.

In the context of clinical practice, several authors have encouraged the inclusion of hearing assessment in memory clinics, or of cognitive screening in hearing clinics (24–26). Recent work has also indicated that audiology patients may themselves be amenable to undertaking cognitive screening in audiological practice (27). Moreover, the addition of hearing and cognitive assessment to memory and hearing clinics, respectively, could help improve both the identification of hearing loss in cognitively-impaired patients and the identification of cognitive impairments in hearing-impaired patients. The latter is of particular importance, as many cognitive assessments have historically been verbally-loaded, resulting in poorer performance in those with hearing loss (28, 29).

Accordingly, it would seem vital that Allied Hearing Healthcare Professionals (AHHPs; e.g., audiologists, audiometrists, etc.) be proficient in some forms of simple cognitive assessment. In Australia currently, AHHPs provide diagnostic assessments across audiological, neurological, and rehabilitation services, which include providing hearing-aid prescriptions, fittings, counselling, assistive listening devices, and implantable devices (30). Furthermore, the scope of practice developed by the three Australian practitioner professional bodies stipulates that AHHPs undertake assessment of patients’ cognitive function and adapt test procedures to patients with complex cognitive needs (30). Similar stipulations have been made internationally, such as with the American Speech-Language-Hearing Association’s requirement that AHHPs screen for cognitive disorders and undertake case-finding for dementia (31).

However, the above stipulations are only prescriptive; that is, they have not addressed the feasibility and acceptance of such cognitive testing within audiological practice and not been supported with the provision of any training or educational programs. Furthermore, there is limited literature on the knowledge, attitudes, and practices of AHHPs’ use of cognitive screening assessments or their understanding of the association between hearing loss and cognitive impairment. In a UK-based study, Leroi et al. (32) investigated Allied-Health professionals across three main specialties (memory clinicians, optometrists, & audiologists), namely through a focus group and Knowledge-Attitude-Practice survey (KAP). Results showed that all specialties valued interdisciplinary assessment and collaboration, due to the high comorbidity of sensory and cognitive disorders in their respective patient populations; they also agreed on the need for interdisciplinary collaboration to develop new screening assessments for patients affected by comorbid sensory and cognitive impairments. However, results also demonstrated that there was low confidence within each specialty in undertaking assessments from other disciplines. An equivalent study has not been conducted in Australia.

The current study therefore aimed to assess Australian AHHP’s knowledge, attitudes, and practices relevant to assessing comorbid hearing loss and cognitive impairment, with the further aim of consequently informing optimal healthcare services for patients with comorbid cognitive impairment and hearing loss in the future. An online self-report KAP survey was developed to be suitable for Australian AHHPs. The knowledge section of the KAP survey generally asked what AHHPs knew about the effect of cognitive impairment on their patients and practice, as well as the administration of cognitive screening tests and referral pathways. The attitude section, meanwhile, asked about AHHPs’ attitudes towards their role in identifying cognitive impairment, administering screening tests, referring patients with possible cognitive impairment, and the challenges related to these factors. Finally, the practice section asked about whether AHHPs were discussing the link between hearing loss and cognitive impairment with their patients, conducting cognitive screening tests, and making forward referrals for medical assessment and management.

## 2 Materials and methods

### 2.1 Study design

This study used a cross-sectional design. A KAP survey developed for Australian AHHPs was used to elucidate their knowledge, attitude, and practices regarding the provision of care for hearing-impaired older adults with suspected cognitive impairment. Ethics approval for this project was received from the University of Western Australia (reference no: 2021/ET000434).

### 2.2 The KAP questionnaire

#### 2.2.1 Development and contents

All survey questions were developed based on discussions with the project team, consisting of a psychologist, a general practitioner, audiologists from Ear Science Institute Australia’s Lions Hearing Clinics, geriatricians, and geriatric psychiatrists from Western Australia Centre for Health and Ageing. The questionnaire consisted of five sections: demographic information, knowledge, attitude, practice, and training (see full questionnaire in Supplementary material).^1^ The demographic section contained questions about the respondent’s sex, ethnic or cultural background, country of residence, years in profession, and audiology-specific qualifications. The knowledge, attitudes, and practice sections included questions on the respondent’s awareness, views, and practice regarding the delivery of hearing-healthcare services to patients with potential cognitive impairment. Meanwhile, the training section included ranked questions on the format and content of training resources desirable to the respondents. Most questions were answered using a 5-point Likert scale (e.g., “Managing clients with cognitive impairment can be challenging” – *strongly disagree* [0], *disagree* [1]*, neutral* [2]*, agree* [3]*, strongly agree* [4]; “I have used formal cognitive screening tests as part of my practice” – *never* [0], *rarely* [1], *occasionally* [2], *frequently* [3], *very frequently* [4]), though some questions were either binary (e.g., “I have used formal cognitive screening tests as part of my practice” – *no* [0], *yes* [1]) or multiple-choice (e.g., “I decide to do a cognitive screening test on older clients based on: [choose all that apply]” – a. client’s age; b. client reporting memory issues; c. carer/family reporting memory issues; d. inconsistent hearing assessment results; e. other); some questions also allowed for open-ended elaboration (e.g., “Please describe how you decide to conduct a cognitive screening test”). Lastly, ranked questions were used in the training section (e.g., “Please indicate your preference [with 1 = first preference, 4 = last preference] for the kind of training that would help to empower you to work with clients with hearing loss and cognitive impairment:” – __ online course/workshop; __ in-person course/workshop; __ book/journal article; __ clinical guidelines/tip sheets).

After initial development, the survey was reviewed by a focus group of approximately ten audiologists from Lions Hearing clinics (Western Australia). Upon providing written informed consent, a 90 min facilitated discussion took place, with participant responses being recorded in a written log. Participants first shared their general reflection about the whole survey, and then addressed individual questions in terms of their clarity and usefulness. Relevant questions were subsequently revised according to the focus group’s feedback. Finally, five audiologists pilot-tested the survey to assist in eliminating issues, which included verifying feasibility regarding survey length, layout across different devices, and ease of completion.

#### 2.2.2 Participants and survey delivery

The survey was sent via the Qualtrics survey platform (Qualtrics, Provo, UT) to email accounts of currently practising, registered members of Audioloy Australia (AudA), the Australian College of Audiology (ACAud), and Hearing Aid Audiometrist Society of Australia (HAASA), which collectively form the main hearing-healthcare professional bodies in Australia—though, note that registration is not compulsory to practice. The total number of AHHPs who received an invitation email was estimated to be approximately 4,000, which comprised approximately 3,000 AudA members, 816 ACAud members, and 141 HAASA members.

Emails invited recipients to participate in the survey and provided a hyperlink. Participants were required to firstly read a participant information form, and then to provide informed consent if they wished to proceed with the survey. A total of two weeks was given for participants to complete the survey online, with a reminder being sent a week before the survey closed. Once started, the survey had no time limit. For data to be included in analyses, participants had to have: (1) provided informed consent; (2) been living in Australia; and (3) completed more than 20% of the survey.

### 2.3 Data analysis

Data were visualised using Python (Version 3.10.5, Python Software Foundation) and analysed using SAS software (Version 9.4, copyright © 2016 by SAS Institute Inc., Cary, NC, United States). Frequencies and percentages are provided for demographic data (sex, ethnicity, experience, and qualification), as well as each Likert item in the knowledge (all except K12), attitude (all except A7 & A8), and practice (all except P4, P5, P11 & P12) sections; multiple-choice items are presented in bar-graph format, while dichotomous-choice items are discussed in-text. Responses to open-ended items (i.e., P4b, P5b, P7a, P12a, and T1a) are provided in the Supplementary material (see footnote 1); note that there were few responses to these items. Likert-item responses are also presented graphically to demonstrate the balance of agreement across items. In order to determine whether odds of agreement for each Likert item statistically differed between categories of the demographic variables (e.g., sex: male vs. female), binary logistic regression was performed; accordingly, Likert-scale data were dichotomised into positive (i.e., “*strongly agree*” to “*agree*”; “*very frequently*” to “*frequently*”; “*always*” to “*very often*”) and negative (i.e., “s*trongly disagree*” to “*neutral*”; “*never*” to “*occasionally*”; “*never*” to “*sometimes*”) categories. These binary logistic regression analyses were performed at the item level, as the items did not form distinct knowledge, attitude, and practice factors; please see the Supplementary material (see footnote 1) for a report of the exploratory factor analysis performed on our data. The Firth method was used in instances of quasi-complete separation (i.e., knowledge questions 1, 2, 6, and 12; attitude questions 1, 3, and 6; and practice questions 3 to 6). Odds ratios (*ORs*), 95% confidence intervals (95% *CIs*) and *p*-values are provided. Statistical significance was considered at the 5% level. Power calculation indicated a sample size of 351, given a population of 4,000, confidence level of 95%, and margin of error of 5%.

## 3 Results

### 3.1 Participants’ demographic information

Of 4,000 invitations, we received 117 responses (response rate of ~2.9%), with 101 meeting inclusion criteria (~ 2.5% of initial invitations). Note that two participants who did not specify their sex were excluded from analysis, as this group size was not large enough to be analysed. In addition, 15 participants failed to complete the entire survey, with 10 of these participants failing to complete over 20%; further, two participants were outside Australia, one did not consent to complete the survey, and two responses were undeleted test previews. Consequently, after taking all exclusions and inclusion criteria into account, sample sizes of our analyses ranged between *N* = 101 and *N* = 85 across items. As shown in Table 1, most participants in the final sample were female, identified as Caucasian, held a postgraduate qualification in audiology, and possessed more than 10 years of experience working in the field.

**Table 1:** Demographics of survey participants.

Demographics	Number (%)
Sex	
Female	77 (76.23%)
Male	24 (23.77%)
Ethnicity	
Caucasian	68 (67.33%)
Asian	16 (15.84%)
European	10 (9.90%)
Other	7 (6.93%)
Qualification	
Postgraduate^*^	64 (63.37%)
Bachelor Degree^†^	14 (13.86%)
Diploma/Certificate^†^	23 (22.77%)
Years’ experience	
>10	64 (63.37%)
5–10	20 (19.80%)
2–5	9 (8.91%)
<2	8 (7.92%)

*Masters or Doctorate, † or equivalent qualification.

### 3.2 Knowledge

#### 3.2.1 Descriptives of knowledge items

**Figure 1:**
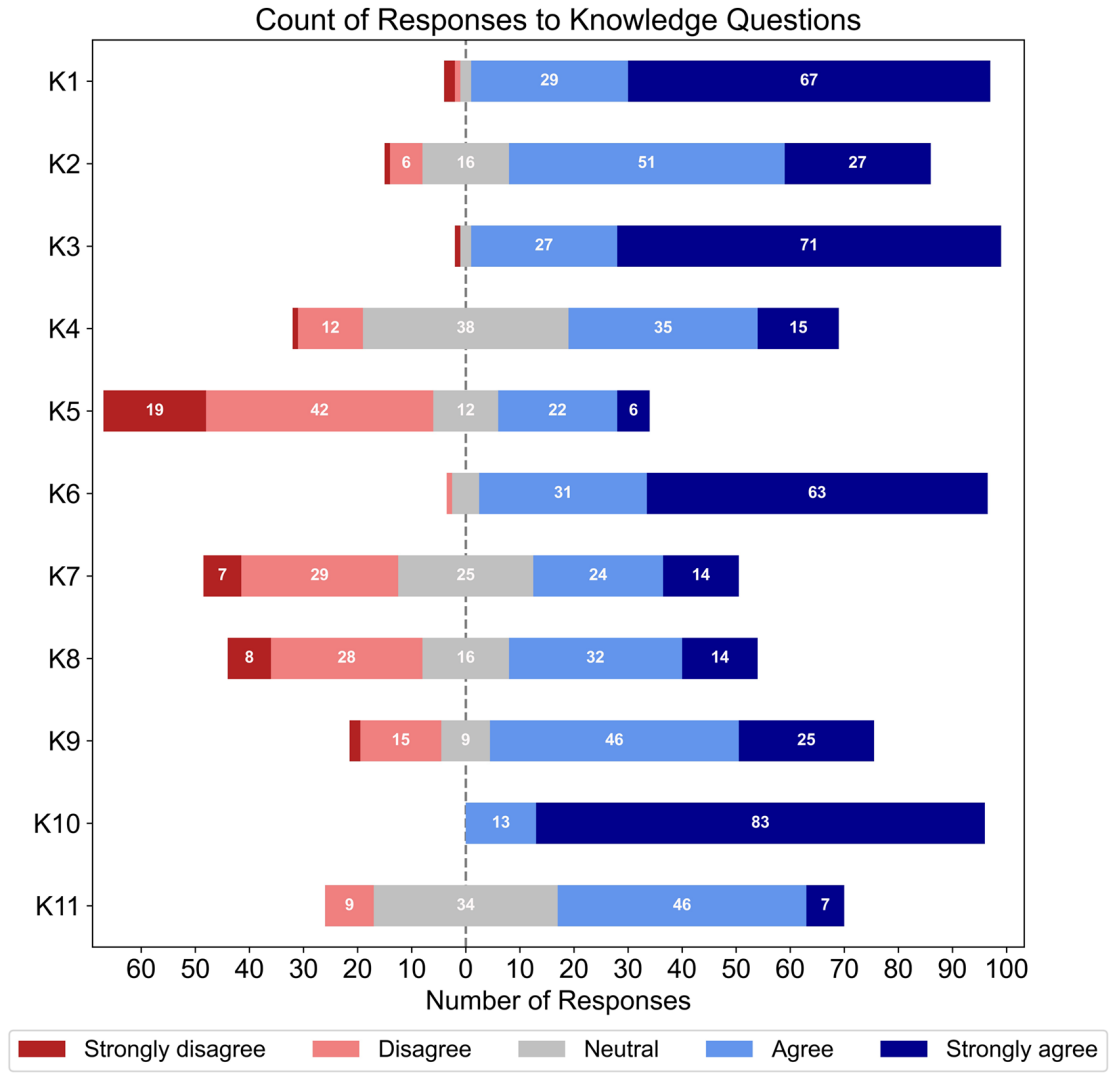
Frequencies of responses to each Likert-based item in the knowledge section; note that the total number of responses for each item varied (K1 – K5, *N* = 101; K6, *N* = 100; Q7, *N* = 99; K8, *N* = 98; K9, *N* = 97; and K10 – K12, *N* = 96).

As seen in Figure 1, respondents generally showed high awareness of the potential comorbidity between hearing loss and cognitive impairment (K1, *N* = 101), the existence of objective hearing loss assessments for patients with cognitive impairments (K2, *N* = 101), and the need to increase clinic session time and provide alternative care options for comorbid patients (K3, *N* = 101). High awareness was also seen for the need to provide instructions for hearing-aid use in written/visual forms (K6, *N* = 100), how to initiate referral pathways for comorbid patients requiring further hearing loss assessment (K9, *N* = 97), and the need to obtain valuable information through family/carers and their attendance at clinic sessions (K10, *N* = 96).

Conversely, respondents’ awareness was mixed for cognitive screening tests that account for hearing loss (K4, *N* = 101), how to incorporate cognitive-support needs in hearing rehabilitation (K7, *N* = 99), and referral pathways for comorbid patients requiring additional cognitive assessment (K8, *N* = 98). Respondents also had more mixed awareness of how to accurately identify cognitive impairments (K11, *N* = 96), and most respondents disagreed that they had the training and expertise to administer cognitive screening tests (K5, *N* = 101). Finally, this section’s binary-choice question showed that two-thirds of participants (66.7%) were unaware that all Australian adults over 75 years old were administered a cognitive screening test by their GP (K12, *N* = 96).

#### 3.2.2 Binary logistic regression of knowledge items and demographic variables

Binary logistic regression with sex as the predictor identified that females were significantly less likely to agree than males (19.4 and 54.1% respectively) that their training was sufficient to administer and interpret a cognitive screening test (K5; *OR* = 0.20, 95% *CI* [0.07, 0.54], *p* < 0.002, *N* = 101, *df* = 1). Similarly, females were less likely than males (30.6 and 62.5% respectively) to agree that they knew how to incorporate structured cognitive support needs in hearing rehabilitation (K7; *OR* = 0.26, 95% *CI* [0.10, 0.69], *p* < 0.007, *N* = 99, *df* = 1). Females were also significantly less aware than males (25.3 and 61.9% respectively) that all adults in Australia over the age of 75 are administered a cognitive screening test by their GP (K12; *OR* = 0.22, 95% *CI* [0.08, 0.6], *p* < 0.004, *N* = 96, *df* = 1).

Further binary logistic regressions with qualification as the predictor showed that those with a bachelor’s degree or equivalent were less likely to agree than those with postgraduate qualifications (78.5 and 95.3% respectively) that hearing-device instructions for those with cognitive impairment should be supplemented with written/visual forms (K6; *OR* = 0.18, 95% CI [0.03, 0.97], *p* = 0.046, *N* = 100, *df* = 1). Lastly, binary logistic regression using ethnicity as a predictor showed that Asian participants were significantly more likely (73.3%) than those of other ethnicities (14.2%) to indicate awareness of how to initiate formal referral pathways for comorbid patients who need further assessment of their memory (K8; *OR* = 16.49, 95% CI [1.48, 182.91], *p* < 0.023, *N* = 98, *df* = 1).

Two additional findings of interest marginally failed to meet statistical significance in our binary logistic regressions with qualification and years of experience as predictors, respectively. For the former, those with a bachelor’s degree were less likely (85.7%) than those with a postgraduate degree (98.4%) to know that clients with cognitive impairments require more time and alternative tests (K3; *OR* = 0.11, 95% *CI* [0.0, 1.01], *p* < 0.052, *N* = 101, *df* = 1). For the latter, participants with 5 to 10 years’ experience were less likely to know (22.2%) than those with over 10 years’ experience (48.4%) how to incorporate structural cognitive support needs in their practice (K7; *OR* = 0.30, 95% *CI* [0.09, 1.02], *p* < 0.055, *N* = 99, *df* = 1). All other binary logistic regressions with knowledge items were non-significant.

### 3.3 Attitude

#### 3.3.1 Descriptives of attitude items

**Figure 2:**
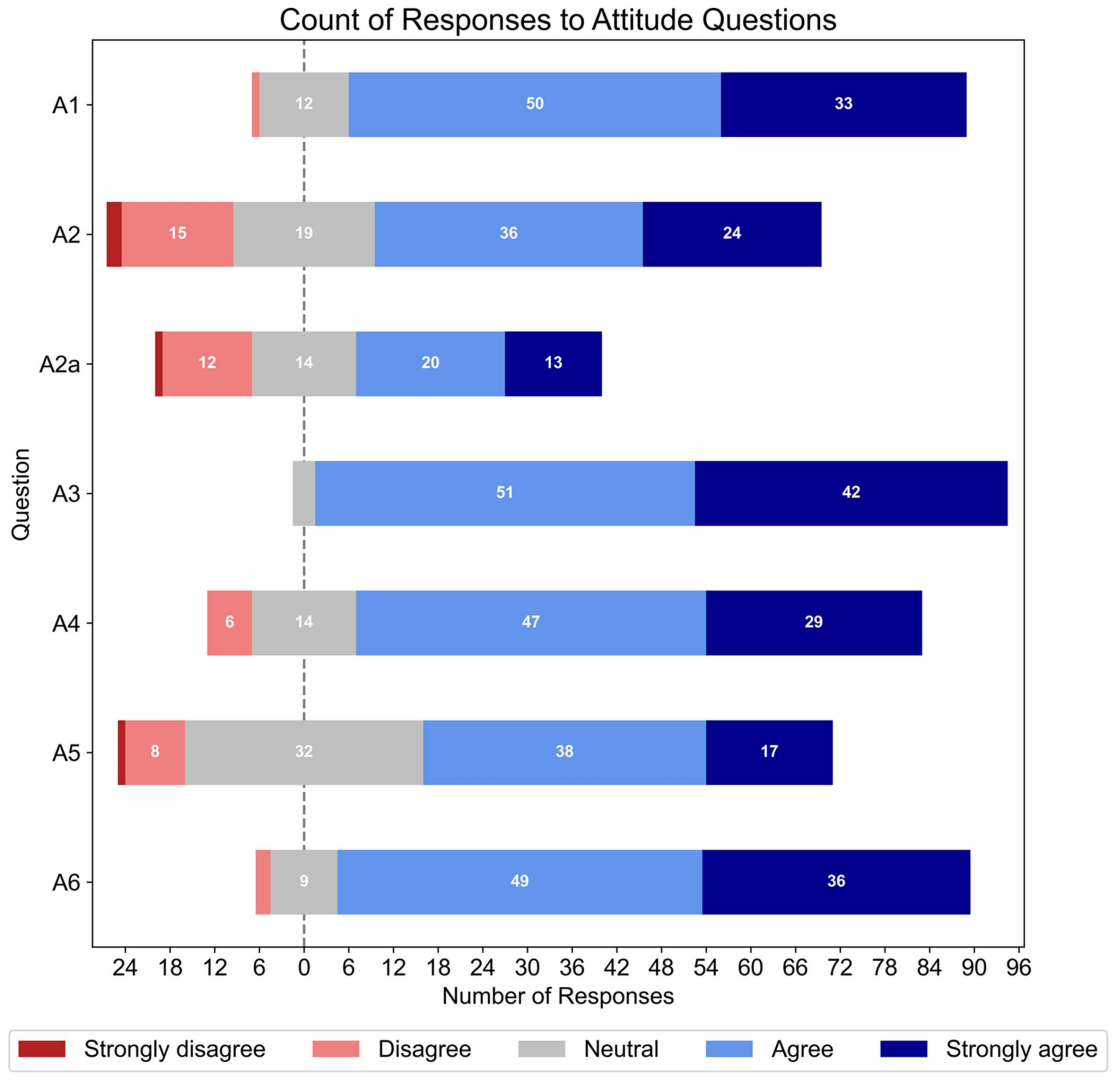
Frequencies of responses to each Likert-based item in the attitude section. The total number of responses for each item varied (A1 – A6, *N* = 96; however, Q2a, *n* = 60).

Figure 2 shows the percentage of responses for each Likert item within the attitude section of the survey. Strong agreement was found for the perceived value of asking patients about memory issues (A1, *N* = 96), difficulty of managing patients with cognitive impairments (A3, *N* = 96), and role of AHHPs in identifying cognitive impairments in patients with hearing loss (A4, *N* = 96); there was also high agreement that AHHPs should refer patients with cognitive impairments to other health professionals for follow-ups (A6, *N* = 96). Respondents had more split agreement in their confidence to ask older patients if they had memory issues (A2, *N* = 96); those who were more confident then showed split agreement on their confidence to have an in-depth discussion with patients about their memory issues (follow-up question A2a, *n* = 60). Mixed agreement was also found for the appropriateness of AHHPs administering cognitive screening tests to patients with hearing loss (A5, *N* = 96).

**Figure 3:**
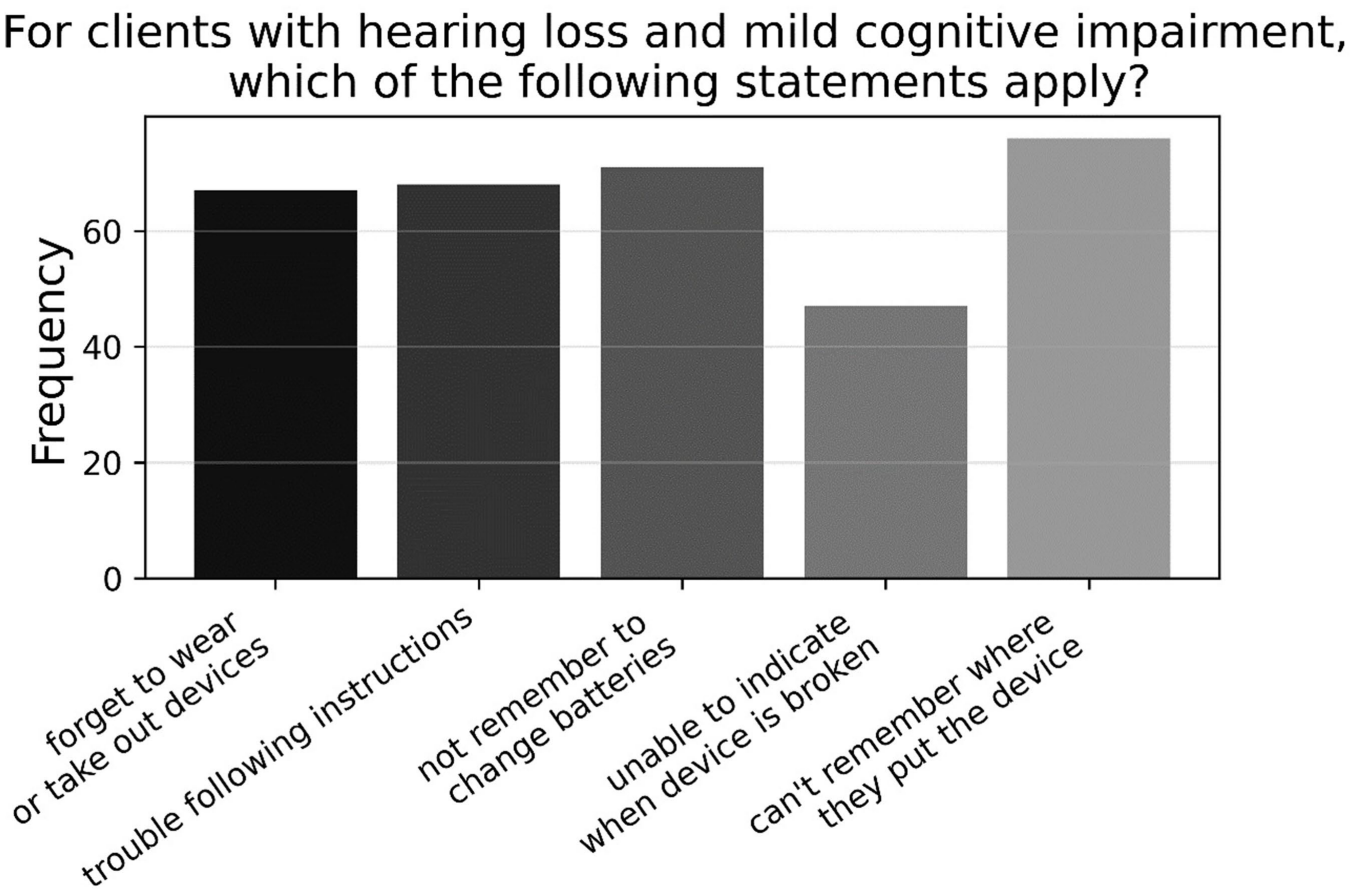
Frequencies of responses to multiple-choice-based item A7 in the attitude section (*N* = 95).

**Figure 4:**
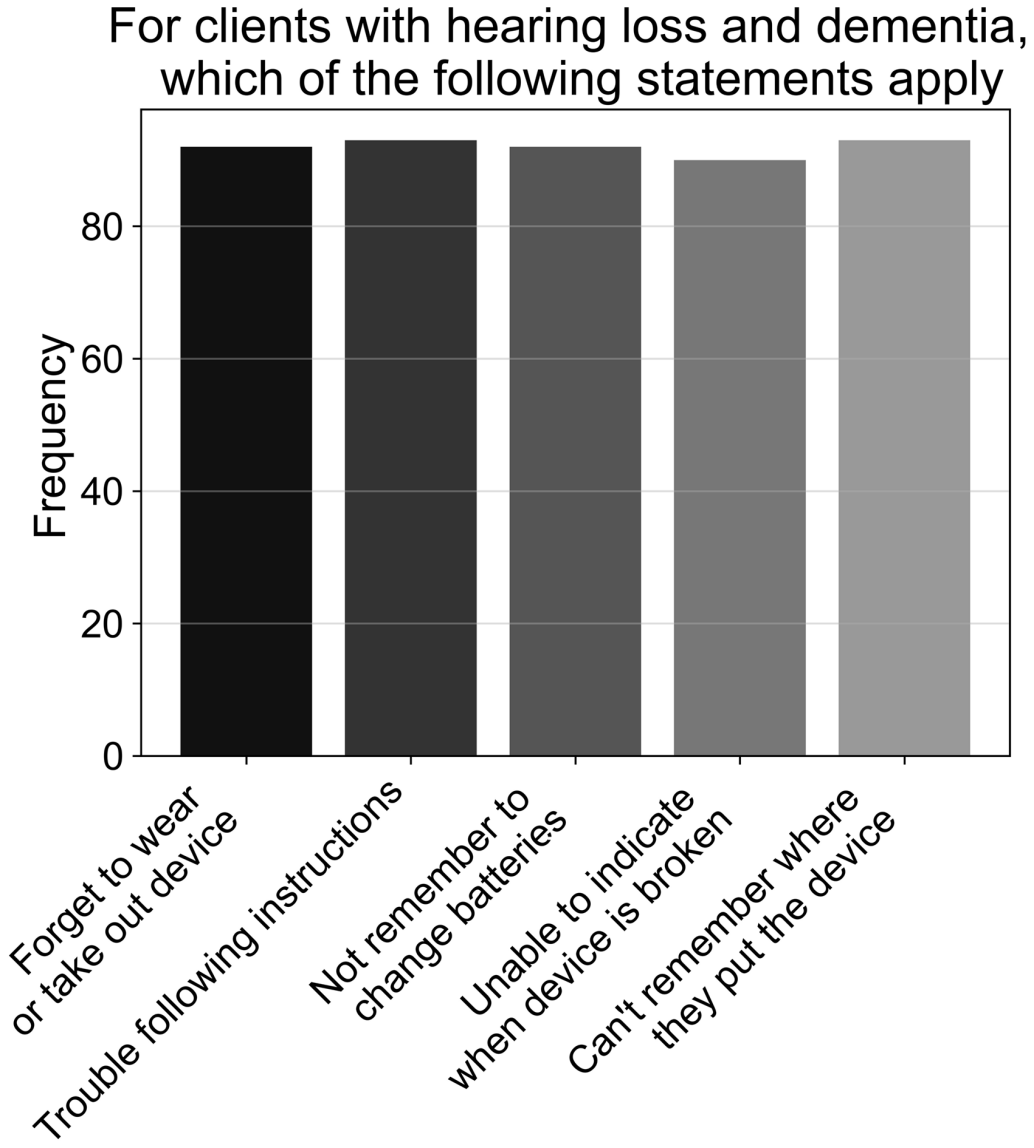
Frequencies of responses to multiple-choice-based item A8 in the attitude section (*N* = 95).

As shown in Figure 3, when listing reasons why patients with hearing loss and MCI may experience challenges in using hearing aids (A7, *N* = 95), just over two-thirds of participants (69.2%) listed all reasons; consisting of memory deficits (i.e., forgetting to use or take out device; changing the batteries; misplacing device), and cognitive issues (i.e., unable to indicate if device is broken; trouble following instructions during a clinic session). The remaining half of respondents gave an approximately equal amount of responses for other combinations of the options provided, with most having memory deficits included in their answers. However, when rephrased to ask what reasons patients with hearing loss and dementia may experience challenges in using hearing aids (A8, *N* = 95), 96.8% listed all reasons specified above—see Figure 4.

#### 3.3.2 Binary logistic regression of attitude items and demographic variables

Binary logistic regression with sex as the predictor found that females were less confident (56%) than males (85.7%) to ask patients if they had memory issues (A2; *OR* = 0.21, 95% CI [0.05, 0.78], *p* < 0.020, *N* = 96, *df* = 1). Further binary logistic regressions with qualification as the predictor showed that those with a bachelor degree agreed less (64.2%) than those with a postgraduate degree (90.1%) about the value of asking patients about their memory during assessments (A1; *OR* = 0.20, 95% *CI* [0.05, 0.78], *p* < 0.022, *N* = 96, *df* = 1). Participants with a bachelor’s degree were also less likely to agree (57.1%) than participants with a postgraduate qualification (83.6%) that AHHPs have a role to play in identifying cognitive impairments in those with hearing loss (A4; *OR* = 0.26, 95% *CI* [0.07–0.91], *p* < 0.037, *N* = 96, *df* = 1). All other binary logistic regressions with attitude items were non-significant.

### 3.4 Practice

#### 3.4.1 Descriptives of practice items

**Figure 5:**
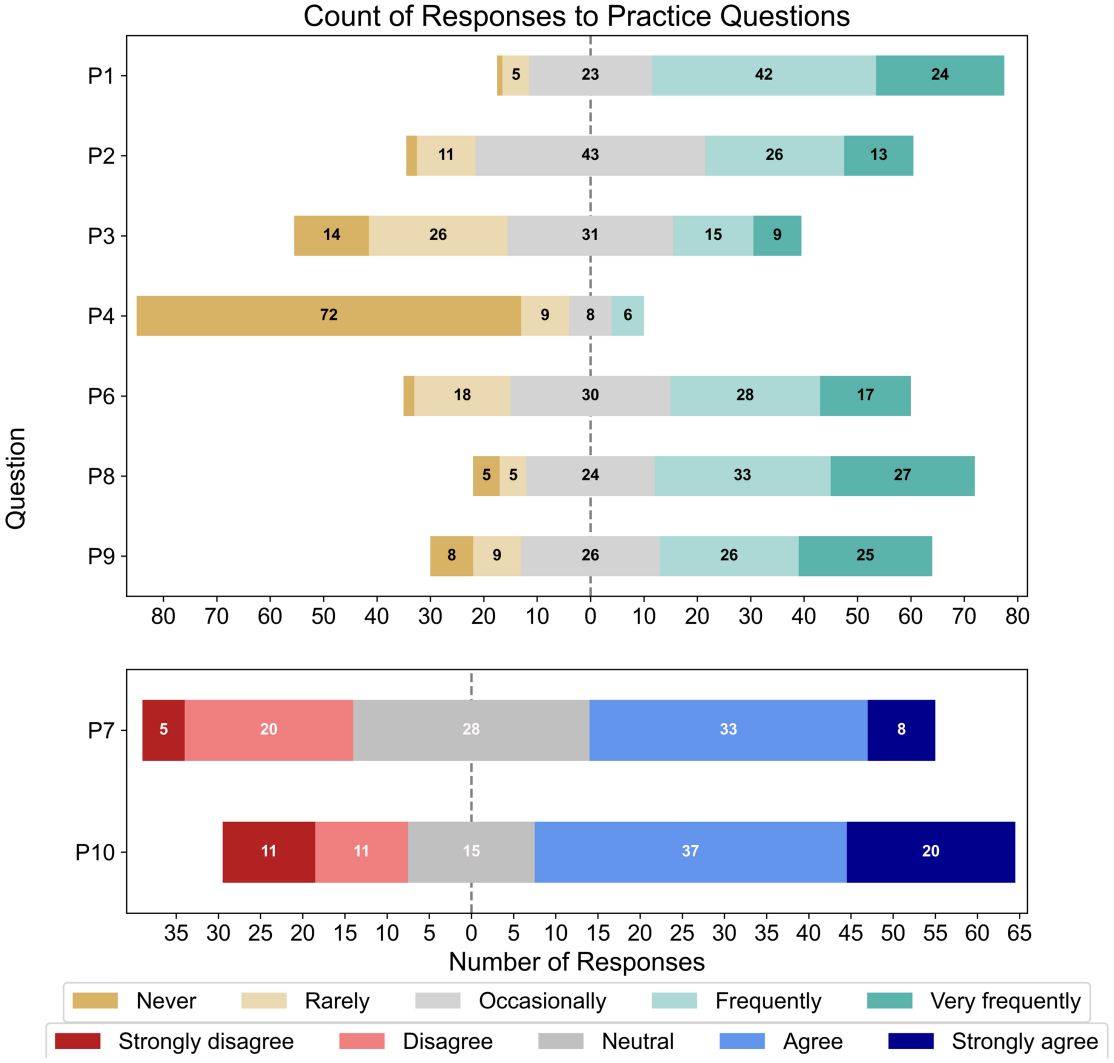
Frequencies of responses to each Likert-based item in the practice section; note that the total number of responses for each item varied (P1 – P4, *N* = 95; P6 – P10, *N* = 95).

Figure 5 shows the percentage of responses for each Likert item within the practice section of the survey. Over two-thirds of respondents spoke to their clients about the association between hearing loss and cognitive impairment, with approximately a quarter doing so sometimes (P1, *N* = 95). Most respondents did not conduct cognitive screening as a part of their practice (P4, *N* = 95) and most occasionally or rarely recommended objective hearing assessments if they suspected a patient’s cognitive impairment affected their hearing loss tests (P3, *N* = 95). Approximately half of the respondents frequently/very frequently talked to patients about how their cognitive impairment could impact their hearing rehabilitation (P6, *N* = 95), and allocated extra time in sessions for patients with suspected comorbidity (P9, *N* = 94). Further, approximately two-thirds of respondents provided instructions for hearing-device use in written or video formats for comorbid patients (P8, *N* = 94). Meanwhile, under half (45.3%) of respondents occasionally asked patients or family/carers about their patient’s cognitive functioning (P2, *N* = 95), with 41.2% doing so frequently/very frequently. When asked about having effective tools to help comorbid patients use hearing devices (P7, *N* = 94), 26% strongly disagreed/disagreed, 30.2% were neutral, and 43.7% agreed/strongly agreed. Just over half of respondents also agreed/strongly agreed (59.3%) that their workplace allowed them extra session time to support suspected comorbid patients (P10, *N* = 94), while 23.9% disagreed/strongly disagreed.

**Figure 6:**
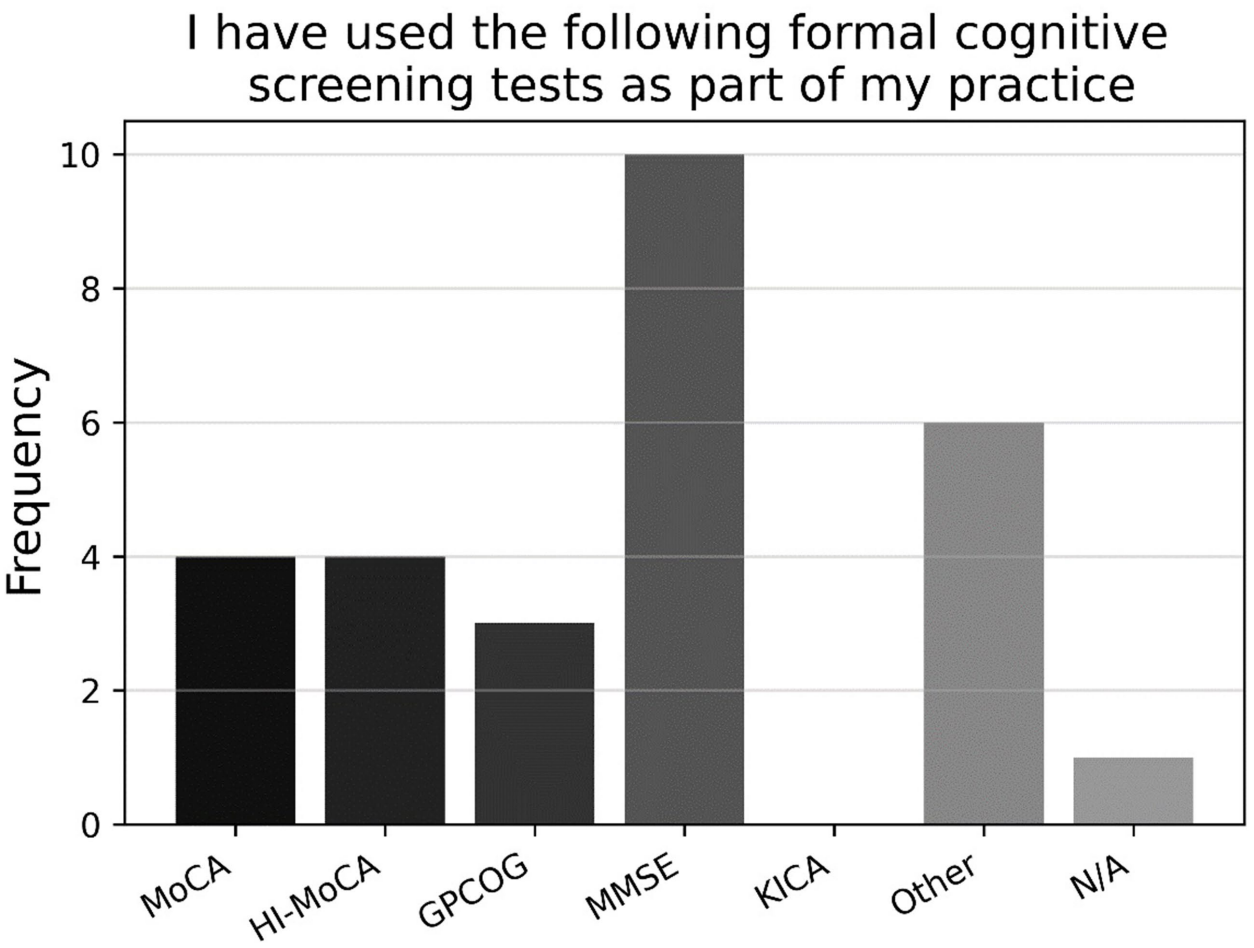
Frequencies of responses to multiple-choice-based item P5a in the attitude section (*n* = 21).

**Figure 7:**
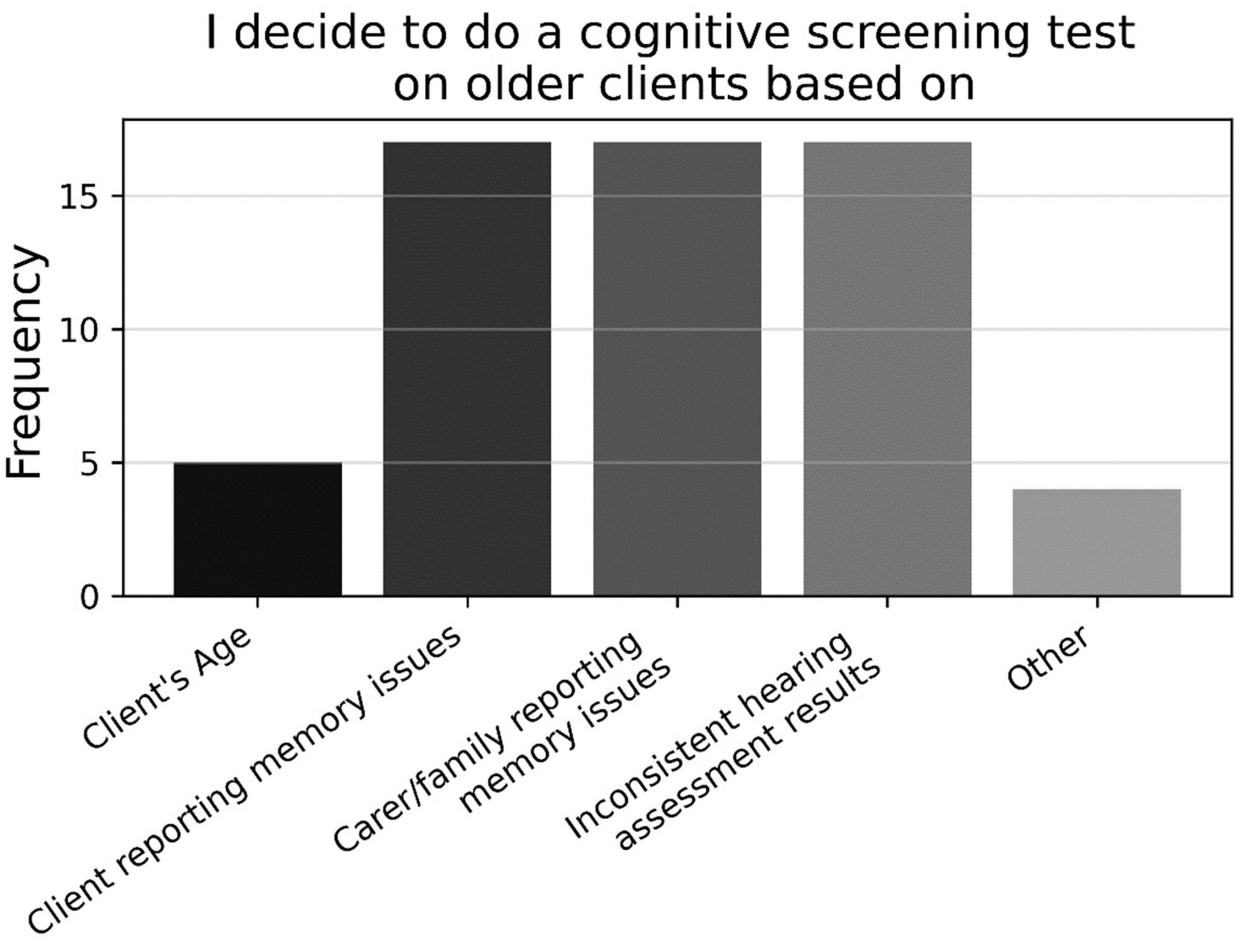
Frequencies of responses to multiple-choice-based item P4a in the practice section (*n* = 23).

Looking at the binary and multiple-choice questions, most respondents had never used a formal cognitive test previously in their practice (77.9%; P5, *N* = 95). As shown in Figure 6, those who had previously used a formal cognitive test had mostly used the MMSE, followed by the (Hi-) MOCA, and GPCOG, with a large proportion using some other task not listed (P5a, *n* = 21). As shown in Figure 7, those who had previously used cognitive screening tests usually did so based on subjective memory complaints from the client or family/carer, or inconsistent hearing-assessment results; less common was the use of cognitive screening tests based on client age, or some unspecified alternative (P4a, *n* = 23).

**Figure 8:**
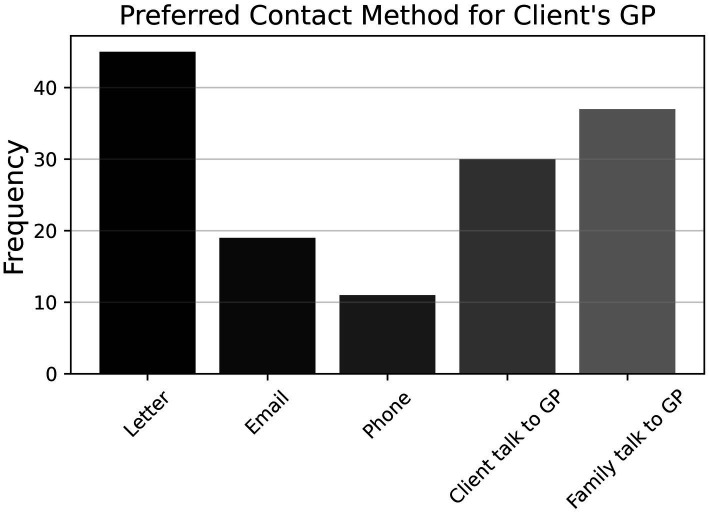
Frequencies of responses to multiple-choice-based item P11a in the practice section (*n* = 54).

**Figure 9:**
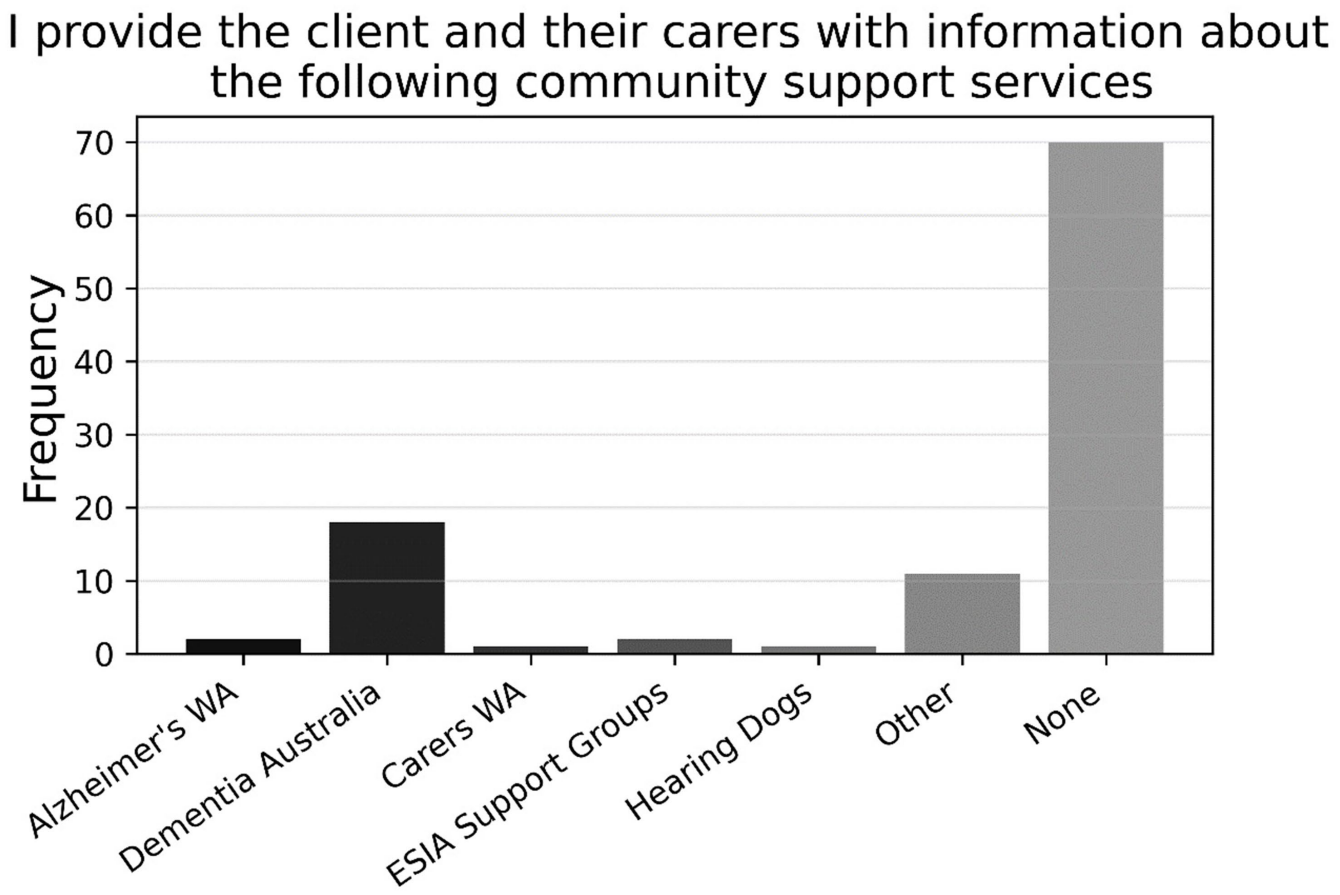
Frequencies of responses to multiple-choice-based item P12 in the practice section (*N* = 94).

Further, there was a close split on item P11 (*N* = 94) between respondents who engaged with patients’ GPs if they suspected cognitive impairment (57.2%) and those who did not (42.7%). Figure 8 shows that the former preferred to contact GPs by letter, followed by requesting family/carer to contact GP, requesting client to contact GP, email, and phone (P11a, *n* = 54). Finally, as shown in Figure 9, approximately three-quarters of participants (74.5%) indicated that they did not refer any clients to community support services for cognitive impairment (P12, *N* = 94); for those who did refer to such services, Dementia Australia was most popular, followed by Alzheimer’s WA and Carers WA, then ESIA Support Groups and Hearing Dogs—eleven respondents referred to some other, unspecified service.

#### 3.4.2 Binary logistic regression of practice items and demographic variables

Binary logistic regression with sex as the predictor showed that females were less likely than males (16.2 and 42.8% respectively) to use formal cognitive screening tests as a part of their practice (P5; *OR* = 0.26, 95% *CI* [0.09, 0.75], *p* < 0.014, *N* = 98, *df* = 1). A further binary logistic regression with qualification as the predictor indicated that participants with a bachelor’s degree were less likely (15.3%) than those with a postgraduate degree (47.5%) to ask patients or their families about a patient’s cognitive status (P2; *OR* = 0.20, 95% *CI* [0.04, 0.98], *p* < 0.050, *N* = 97, *df* = 1). All other binary logistic regressions with practice items were non-significant.

### 3.5 Support received and preference for training

**Figure 10:**
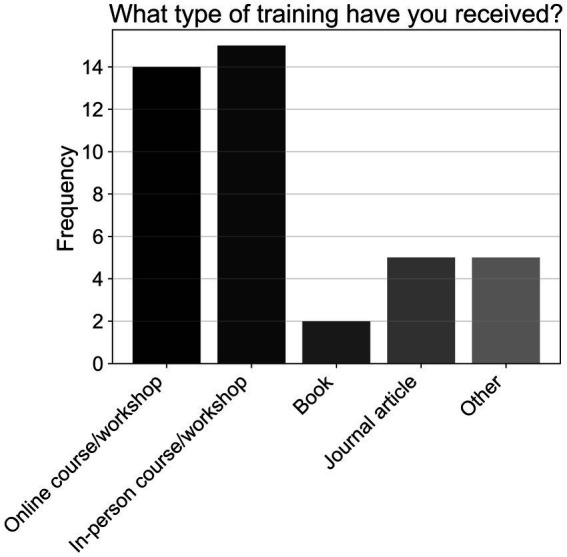
Frequencies of responses to multiple-choice-based item T1a in the practice section (*n* = 24).

Approximately three-quarters of respondents (74.47%; T1, *N* = 96) reported not receiving training to support patients with cognitive impairments. As shown in Figure 10, of those who did receive training, most attended online or in-person workshops, which was followed in popularity by journal articles, unspecified alternative forms of training, and books (T1a, *n* = 24).

**Figure 11:**
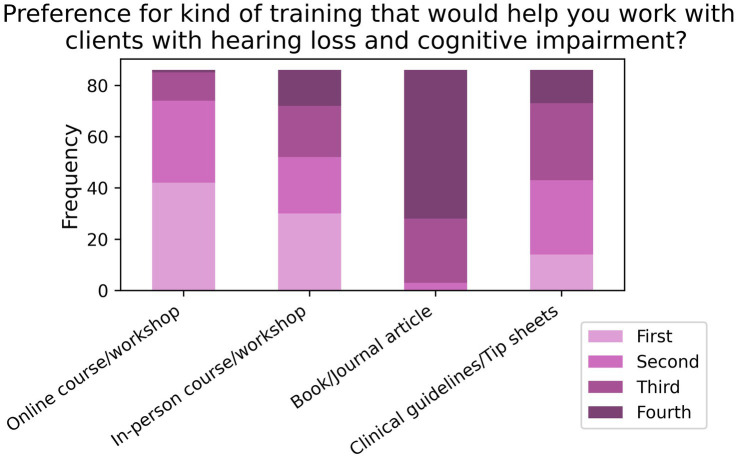
Frequencies of responses to rank-based item T2 in the training section (*N* = 86).

**Figure 12:**
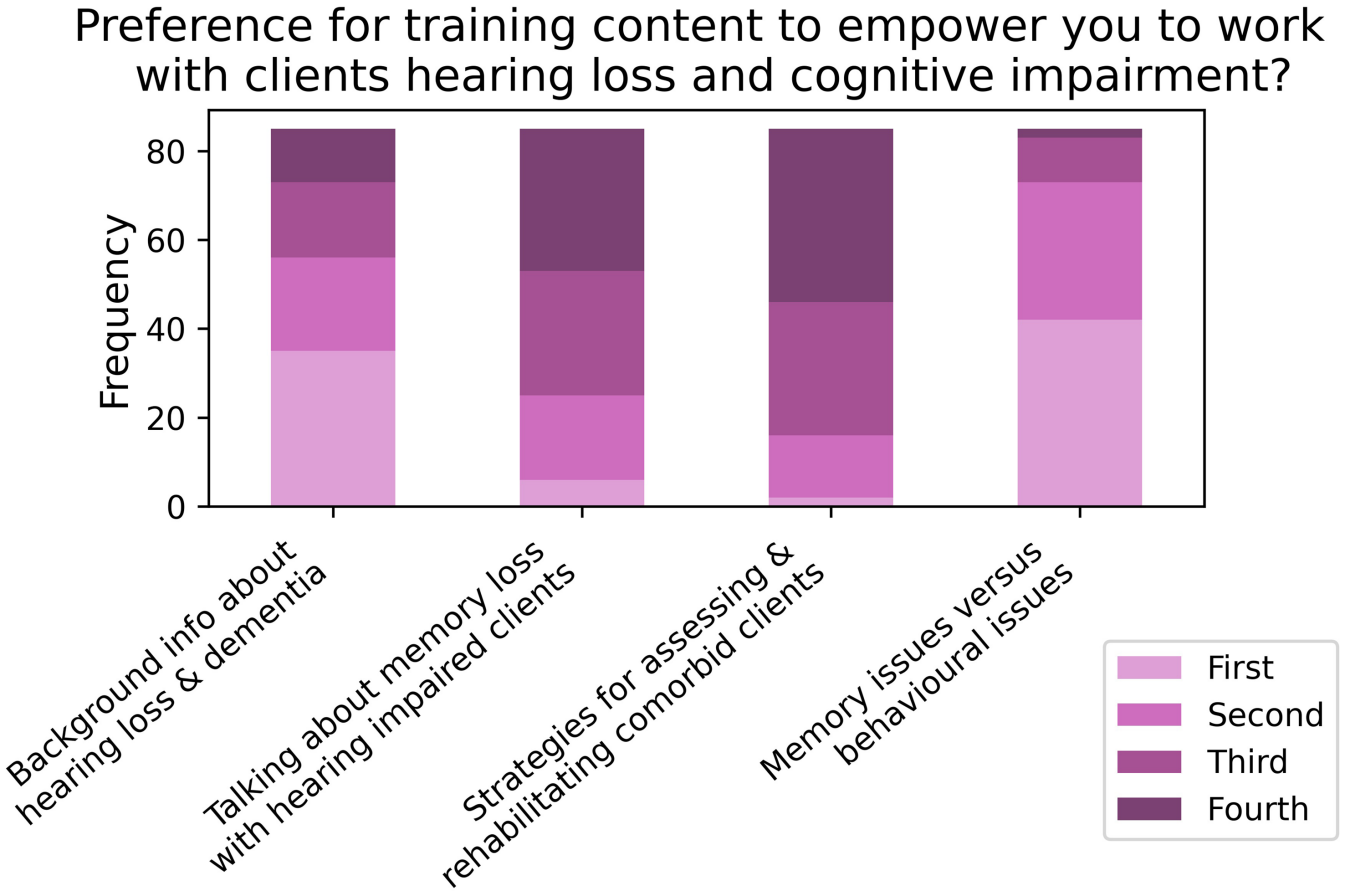
Frequencies of responses to rank-based item T3 in the training section (*N* = 85).

When asked to rank their preference for different forms of training aimed at improving care for comorbid patients (T2; *N* = 86; see Figure 11), 85.2% listed online training as either their first or second preference, followed by 60.2% for in-person training in first or second preference. When asked about what contents to include in the training (T3, *N* = 85; see Figure 12), 81.8% of respondents listed “clinical strategies for assessing and rehabilitating hearing-impaired clients with cognitive impairment” as either their first or second preference, while 70.6% of participants listed “how to talk about memory loss with hearing impaired clients” as their first or second preference.

## 4 Discussion

The current study investigated allied hearing-healthcare professionals’ (AHHPs) knowledge, attitude, and practice in relation to providing services and care for patients with comorbid hearing loss and suspected cognitive impairment. While our sample size (*N* = 101) was lower than that recommended by our power calculation (*N* = 351), our sample was reasonably reflective of the general AHHP population in Australia—that is, mostly female with postgraduate qualifications (33).

Our findings suggest that AHHPs are highly aware of the established link between hearing loss and cognitive impairment. According to our survey, many AHHPs increased consultation time for clients suspected of having comorbid hearing loss and cognitive impairment, spoke about the effects of cognitive impairment on hearing rehabilitation to their clients, and provided instructions for hearing-aid use in visual formats; these suggest that professional standards for treatment modification in the “Scope of Practice for Audiologists and Audiometrists” (30), namely related to cognitive impairment and hearing loss, are generally being upheld.

However, our results further suggest that AHHPs are not confident in performing cognitive assessments, and have limited training to support comorbid patients with cognitive impairment and hearing loss; both findings are consistent with previous reports (32, 34). Many respondents were also uncertain about the existence of cognitive assessments designed to account for hearing loss [e.g., the Hearing-impaired version of the Montreal Cognitive Assessment (HI-MOCA); (35)].

Furthermore, while our results suggest that AHHPs generally valued asking clients about their cognitive status and being further involved in identifying cognitive impairments, their confidence for performing these tasks was low. This lack of confidence and reduced feeling of responsibility to carry out cognitive testing may have reflected respondents’ lack of knowledge and attitudes in other areas of healthcare practice and medical disciplines. For example, there was a relative lack of knowledge about initiating referral pathways for patients requiring further cognitive assessment; further, agreement was lower for the suggestion that AHHPs should be administering cognitive screening tests, which belong to another discipline and may not be generally considered core competency for an AHHP. Another potential contributor to AHHPs’ lack of confidence could be related to the negative effects on patient-clinician interaction of a patient having cognitive impairment. [e.g., (36, 37)]. Indeed, discussion of dementia or mild cognitive impairment can be a highly emotional experience for both patient and clinician. To compound this, communication difficulties due to cognitive impairment (38) could contribute to AHHPs apprehension to probe the subject more deeply with a patient or their family. However, as the current study did not assess AHHPs’ feelings regarding the emotional aspects of engaging with patients with cognitive impairment, further research is needed on AHHPs’ need for training on the emotional aspects of dealing with cognitive impairment.

Moreover, our findings suggest that current practices by AHHPs mainly consist of informal assessment of patients’ cognitive status, primarily through direct questioning of patients and/or their family/carers, rather than formal cognitive testing. While this form of assessment has utility to detect subjective memory complaints and cognitive issues, it is markedly less accurate than formal cognitive screening (39); it may also contribute to underdiagnosis of comorbid MCI and hearing loss in audiology clinics. Indeed, while current practices of informal questioning may be sufficient under present professional standards (30), they are likely insufficient to effectively screen for MCI. Thus, adopting assessments like the Dementia Screening Interview, which have been used to assess MCI (40), may allow for minimal changes to current practices that could improve screening outcomes. For further potential improvements to client outcomes, current standards could be modified to require more-robust cognitive screening tools specific to the hearing impaired [e.g., HI-MoCA; (35)], which could be performed by audiologists or through more-formalised referral pathways established in audiological practice. Future work is required to encourage the adoption of formal, objective cognitive tests in audiological practice. Meanwhile, improving interdisciplinary collaboration with other healthcare disciplines and services involved in caring for patients with comorbid hearing loss and cognitive impairment—such as with general practitioners, geriatricians, and memory clinicians—may help to improve AHHPs’ knowledge and skills in cognitive assessment. Our study also suggests that some AHHPs tend to adopt informal paths of referral (i.e., asking family and carers to take the client directly to a GP themselves) for patients with comorbid cognitive impairment and hearing loss, namely when further medical and cognitive assessment is needed. This finding further suggests that AHHPs require training to improve their confidence in directly referring clients for further assessment to other health professionals, which would facilitate interdisciplinary communication and collaborative care.

Increased training for AHHPs in cognitive assessment and the management of patients with comorbid hearing loss is also essential. Indeed, approximately 60% of AHHP respondents in our survey had not received formal training for performing cognitive assessments. Of note, there was a general trend for female AHHPs to indicate less training, experience, and awareness than male AHHPs for cognitive screening and support issues. Furthermore, female AHHPs rated themselves as less confident to ask patients about memory issues, and to administer cognitive screening tests in audiological sessions. However, due to the subjective nature of our primary measure, it is not possible to know whether these observed sex differences were due to objective differences or simply differences in perception of knowledge and skills by female versus male AHHPs. For instance, male AHHPs may have rated their knowledge and experience higher due to overconfidence. Therefore, further investigation is needed to determine whether the observed sex differences are objectively detectable, namely with behavioural measures, or simply subjective due to differences in confidence; this is especially important when considering that females account for the majority of AHHPs in Australia. Moreover, respondents with postgraduate qualifications were more likely than those with bachelor’s degrees to value and provide services beneficial to comorbid patients; this may simply reflect the different levels of education and occupational responsibilities that each degree confers. Finally, more years in the hearing-healthcare profession did not coincide with improved knowledge, attitudes, or practices relevant to comorbid hearing loss and cognitive impairment. This finding is perhaps surprising, as one may expect knowledge, attitudes, and practices to improve with greater experience. One possible explanation is that, due to the recent increase in research investigating the link between hearing loss and dementia [for recent reviews, see (5, 41)], audiology courses may be placing greater emphasis on cognitive impairment, thus improving the awareness of newer AHHPs. Conversely, more-experienced audiologists may not have sufficiently focused on the issue of cognitive-impairment. Nevertheless, it is clear that both experienced and inexperienced AHHPs are in equal need of training in the area of comorbid hearing loss and cognitive impairment.

With respect to types of training, of those who had received cognitive-assessment training, most had done so through online or in-person courses and workshops; these forms of training were also ranked most desirable for future training to improve in this area. AHHPs also reported the most desirable topics for training as being greater information about behavioural issues related to memory problems, and theories and background information about hearing loss and dementia itself. Thus, there is an urgent need for new cognitive-assessment training programs aimed at AHHPs in Australia, with the current findings providing insight into how such programs should be designed and implemented. The aim of such a programme would be to empower AHHPs in Australia to better understand the link between hearing loss and cognitive impairment, gain confidence in caring for comorbid patients at risk, and facilitate improvement of cognitive screening methods in audiology.

### 4.1 Study limitations

Only 117 AHHPs out of 4,000 invited responded to the survey. Indeed, low web-survey response rates among healthcare professionals is a known problem (42), with response rates seeming to vary by specialty (43). For audiology specifically, response rates of 16 and 8% have been shown in an American (44) and Australian KAP studies (45), respectively. While contributing factors have been explored previously (46), it is unclear what contributed to the current study’s lower-than-expected response rates (2.9%). Due to this low response rate, it is possible that our sample was biased towards AHHPs with high interest in the survey topic, potentially skewing data towards higher degrees of knowledge, positive attitudes, and current practices. However, a greater number of participants with less interest in the topic would have likely only bolstered the current finding that more training in hearing loss and cognitive impairment comorbidity is needed. Furthermore, the low response rate could reflect the challenges that may be faced when attempting to implement better interdisciplinary clinical practices; that is, it would be more difficult to educate and train a hard-to-reach audience. Future research should therefore seek ways of improving clinician engagement in research within clinics. A further limitation, as mentioned previously, is that only 101 AHHPs data were included after exclusion criteria, meaning we were underpowered based on our power analysis (recommended *N* = 351), so may have failed to detect some genuine effects. Moreover, our analyses had to be done at the item level, as exploratory factor analysis (see Supplementary material) showed that our KAP-survey items did not form knowledge, attitude, and practice factors (i.e., mean scores). This outcome is somewhat unsurprising, as our KAP survey was primarily designed to learn about points of interest in audiological practice, rather than to measure knowledge, attitude, and practice with psychometric precision. However, to simplify future analyses, subsequent research should seek to modify the present KAP survey to better isolate knowledge, attitudes, and practices. Finally, it was noted above that future research could seek to include objective measures of KAPs, as the self-report measures used presently could upwardly bias estimates.

## 5 Conclusion

This study investigated AHHPs’ knowledge, attitude, and practices relevant to providing service and care for patients with comorbid hearing loss and cognitive impairment. In summary, AHHPs generally possessed good knowledge of the link between hearing loss and cognitive impairment, and showed generally positive attitudes towards the value and role of AHHPs to support comorbid patients; this was also true of the relevant practices. However, some aspects of knowledge, attitude, and practice demonstrated a need for additional training and support. This finding was bolstered by our observation that training and support aimed at improving service and care for older adult clients with comorbid hearing loss and cognitive impairment has been limited. Consequently, the current findings encourage the development of training and support programs that empower and upskill AHHPs to care for clients with hearing loss and cognitive impairment.

## Data Availability

The datasets presented in this study can be found in online repositories. The names of the repository/repositories and accession number(s) can be found at: https://osf.io/t2vgh/.

## Footnotes

^1^https://osf.io/t2vgh/
