# Supplementary material for: Knowledge, attitudes, and practices of Australian allied hearing-healthcare professionals: survey on comorbid hearing loss and cognitive impairment
Source: Front Med (Lausanne). 2024 Aug 23;11:1412475. doi: 10.3389/fmed.2024.1412475 (PMC11377262; doi:10.3389/fmed.2024.1412475)
Supplement: Supplementary file 1 [file Data_Sheet_1.docx]

**Supplement**

**KAP Questionnaire**

***Demographic Questions***

**D1** Sex:

- Male (1)
- Female (2)
- Other (3)
- Don't wish to disclose (4)

**D2** Ethnic or cultural background:

- Caucasian (1)
- Asian (2)
- European (3)
- Aboriginal and Torres Strait Islanders (4)
- Other ethnic group (5)

**D3** Country you live in: (*open response*)

**D4** Years in the audiology profession:

- Less than 2 (1)
- 2-5 (2)
- 5-10 (3)
- More than 10 (4)

**D5** What are your audiology-specific qualifications? (please tick your highest qualification):

- Postgraduate qualification (master's or doctoral degree) (1)
- Degree or equivalent (2)
- Diploma, certificate or equivalent (3)
- Other (4)

**D5a** Please describe your qualifications: (*open response)*

***Knowledge Questions***

**K1** When working with older adults, I'm mindful that as well as a hearing loss they may also have a cognitive impairment.

- Strongly disagree (1)
- Disagree (2)
- Neutral (3)
- Agree (4)
- Strongly agree (5)

**K2** There are objective hearing assessments that can be used for people who may have cognitive impairment.

- Strongly disagree (1)
- Disagree (2)
- Neutral (3)
- Agree (4)
- Strongly agree (5)

**K3** Assessing someone with a cognitive impairment means I might need to spend more time, and provide alternative testing and extra support to the client.

- Strongly disagree (1)
- Disagree (2)
- Neutral (3)
- Agree (4)
- Strongly agree (5)

**K4** There are specifically designed cognitive screening tests that can be used with people with hearing loss.

- Strongly disagree (1)
- Disagree (2)
- Neutral (3)
- Agree (4)
- Strongly agree (5)

**K5** I have the training and expertise to administer and interpret the results of a brief cognitive screening test.

- Strongly disagree (1)
- Disagree (2)
- Neutral (3)
- Agree (4)
- Strongly agree (5)

**K6** Instructions for how to use hearing devices for people with cognitive impairment should be supplemented by written and/or visual form.

- Strongly disagree (1)
- Disagree (2)
- Neutral (3)
- Agree (4)
- Strongly agree (5)

**K7** I know how to formally incorporate structured cognitive support needs in hearing rehabilitation plans.

- Strongly disagree (1)
- Disagree (2)
- Neutral (3)
- Agree (4)
- Strongly agree (5)

**K8** I'm aware of how to initiate appropriate formal referral pathways for clients who have both hearing loss and cognitive impairment and need further investigation of their memory.

- Strongly disagree (1)
- Disagree (2)
- Neutral (3)
- Agree (4)
- Strongly agree (5)

**K9** I'm aware of how to initiate appropriate formal referral pathways for clients who have both hearing loss and cognitive impairment and need further investigation of their hearing loss.

- Strongly disagree (1)
- Disagree (2)
- Neutral (3)
- Agree (4)
- Strongly agree (5)

**K10** Family members/carers can provide valuable information regarding the person with hearing loss and their cognitive functioning, and there is value in them attending a hearing appointment.

- Strongly disagree (1)
- Disagree (2)
- Neutral (3)
- Agree (4)
- Strongly agree (5)

**K11** I am able to identify whether a client has a cognitive impairment.

- Strongly disagree (1)
- Disagree (2)
- Neutral (3)
- Agree (4)
- Strongly Agree (5)

**K12** I am aware that in Australia, all adults over the age of 75 are administered a formal cognitive screening test by their GP.

- Yes (1)
- No (2)

***Attitude Questions***

**A1** I see value in asking an older client about memory issues as part of my assessment.

- Strongly disagree (1)
- Disagree (2)
- Neutral (3)
- Agree (4)
- Strongly agree (5)

**A2** I feel confident asking an older client if they have memory issues.

- Strongly disagree (1)
- Disagree (2)
- Neutral (3)
- Agree (4)
- Strongly agree (5)

**A2a** I feel confident having an in-depth discussion with an older client about their memory issues.

- Strongly disagree (1)
- Disagree (2)
- Neutral (3)
- Agree (4)
- Strongly agree (5)

**A3** Managing clients with cognitive impairment can be challenging.

- Strongly disagree (1)
- Disagree (2)
- Neutral (3)
- Agree (4)
- Strongly agree (5)

**A4** Allied Hearing-Healthcare Professionals have a role in identifying cognitive impairment in older adults with hearing loss.

- Strongly disagree (1)
- Disagree (2)
- Neutral (3)
- Agree (4)
- Strongly agree (5)

**A5** It is appropriate for Allied Hearing-Healthcare Professionals to administer brief cognitive screening tests for older clients with hearing loss.

- Strongly disagree (1)
- Disagree (2)
- Neutral (3)
- Agree (4)
- Strongly agree (5)

**A6** Allied Hearing-Healthcare Professionals should refer clients with cognitive impairment to other health professionals/services for follow-up if necessary.

- Strongly disagree (1)
- Disagree (2)
- Neutral (3)
- Agree (4)
- Strongly agree (5)

**A7** For clients with hearing loss and mild cognitive impairment, which of the following statements apply? (Choose all that apply)

- They may forget to wear or take out the devices (9)
- They may not remember to change the batteries (10)
- They may be unable to indicate when the device is broken (11)
- They may not remember where they put the device (12)
- They may have trouble following instructions during an appointment (13)

**A8** For clients with hearing loss and dementia, which of the following statements apply? (Choose all that apply)

- They may forget to wear or take out the devices (9)
- They may not remember to change the batteries (10)
- They may be unable to indicate when the device is broken (11)
- They may not remember where they put the device (12)
- They may have trouble following instructions during an appointment (13)

***Practice Questions***

**P1** I talk to older clients about the link between hearing loss and cognitive impairment.

- Never (1)
- Rarely (2)
- Sometimes (3)
- Very Often (4)
- Always (5)

**P2** I specifically ask older clients and/or their carers or family members questions about the client's cognitive functioning.

- Never (1)
- Rarely (2)
- Occasionally (3)
- Frequently (4)
- Very frequently (5)

**P3** I recommend objective hearing assessments if I suspect that a client’s cognitive impairment is affecting their hearing test results.

- Never (1)
- Rarely (2)
- Occasionally (3)
- Frequently (4)
- Very frequently (5)

**P4** I conduct formal cognitive screening tests with older, hearing-impaired clients as part of my practice.

- Never (1)
- Rarely (2)
- Occasionally (3)
- Frequently (4)
- Very frequently (5)

**P4a** I decide to do a cognitive screening test on older clients based on: (Choose all that apply)

- Client's age (1)
- Client reporting memory issues (2)
- Carer/family reporting memory issues (3)
- Inconsistent hearing assessment results (4)
- Other (5)

**P4b** Please describe how you decide to conduct a cognitive screening test: (*open response*)

**P5** I have used formal cognitive screening tests as part of my practice:

- Yes (1)
- No (2)

**P5a** I have used the following formal cognitive screening tests as part of my practice: (Choose all that apply)

- Montreal Cognitive Assessment (MoCA) (1)
- Montreal Cognitive Assessment for the Hearing Impaired (HI-MoCA) (2)
- General Practitioner Assessment of Cognition (GPCOG) (3)
- Mini Mental State Examination (MMSE) (4)
- Kimberley Indigenous Cognitive Assessment (KICA) (5)
- Other (6)
- Not applicable (7)

**P5b** Please specify what cognitive test you use: (*open response*)

**P6** I talk to clients about how their cognitive impairment could impact their hearing rehabilitation.

- Never (1)
- Rarely (2)
- Occasionally (3)
- Frequently (4)
- Very frequently (5)

**P7** I have the tools to effectively assist hearing-impaired clients with cognitive impairment to use hearing devices.

- Strongly disagree (1)
- Disagree (2)
- Neutral (3)
- Agree (4)
- Strongly agree (5)

**P7a** I use the following tools to assist clients who have cognitive impairment: (*open response*)

**P8** For hearing-impaired clients with cognitive impairment, I provide instructions/information about how to use their hearing devices in writing or in a video format (eg. YouTube).

- Never (1)
- Rarely (2)
- Occasionally (3)
- Frequently (4)
- Very frequently (5)

**P9** I allocate extra time in my consultation to support hearing-impaired clients with suspected cognitive impairments.

- Never (1)
- Rarely (2)
- Occasionally (3)
- Frequently (4)
- Very frequently (5)

**P10** My workplace supports me to allocate extra appointment time for seeing a hearing-impaired client with suspected cognitive impairments.

- Strongly disagree (1)
- Disagree (2)
- Neutral (3)
- Agree (4)
- Strongly agree (5)

**P11** If I suspect that a hearing-impaired client has a cognitive impairment as well, I engage with their GP:

- Yes (1)
- No (2)

**P11a** If yes, my preferred methods of engaging with the GP are: (Choose all that apply)

- Letter
- Phone call
- Email
- Asking the client to talk to their GP
- Asking the client's family member/carer to talk to their GP
- None of the above

**P12** If I suspect that a hearing-impaired client has a cognitive impairment, I provide the client and their carers with information about the following community support services that could assist them: (Choose all that apply)

- Alzheimer's WA
- Dementia Australia
- Carers WA
- Ear Science Institute Australia Support Groups
- Hearing Dogs
- Other
- None

**P12a** Please state which community support service you have informed clients about:

***Training Questions***

**T1** Have you received any training on supporting clients with memory issues or cognitive impairment?

- Yes (1)
- No (2)

**T1a** What kind of training have you attended? (Choose all that apply)

- Online course/workshop
- In-person course/workshop
- Book
- Journal article
- Other

**T1b** Please specify what training you have attended: (*open response*)

**T2** Please indicate your preference (with 1= first preference, 4= last preference) for the kind of training that would help to empower you to work with clients with hearing loss and cognitive impairment:

______ Online course/workshop (including role play and video examples)

______ In-person course/workshop (including role play and video examples)

______ Book/Journal article

______ Clinical guidelines/Tip sheets

**T3** Please indicate your preference (with 1=first preference, 4= last preference) for the content that you would like covered in training, to empower you to work with clients hearing loss and cognitive impairment:

______ Theories and background information about hearing loss and dementia

______ How to talk about memory loss with hearing impaired clients

______ Clinical practice strategies for assessing and rehabilitating hearing-impaired clients with cognitive impairment

______ Memory issues versus behavioural issues

**Responses to Open-Ended Items**

**Table S1.** *Responses given for each of the open-response items.*

| **Open-Response Item** | **Responses** |
| --- | --- |
| D5a | No responses |
| P4b | “Standardised measures of attention, memory and intelligence (screening)”  “problem solving issues, planning issues, learning new information issues”  “Lines of questioning, responses, answers provided”  “if instructed to as part of test protocol” |
| P5b | “The "Clock" test, as screener only!”  “RUDAS”  “MoCA”  “MiniCog”  “Mini ACE”  “Memory subtests of Test of Auditory Processing Skills (v4), Auditory component of Test of Variables of Attention, Kaufmann Brief Intelligence Test” |
| P7a | “written instructions, additional follow ups”  “Written”  “visuals/instructions”  “simplified instructions, care facility co-ordination and maintenance of devices including insertion and removal, multiple follow up aftercare appointments, establish a routine, recommend instruction manual use.”  “Only writing things down or providing written information”  “None”  “none”  “Nil”  “N/A”  “Mmse”  “I draw for them and rephrase if needed until they became confident.”  “Don't have any tools currently”  “Counselling” |
| P12a | “Wicking Centre UTAS”  “Tinnitus Help”  “Just their GP”  “https://www.dementia.org.au/states/nsw”  “GP Community Health Qld Health memory clinicS”  “Dementia Friendly Community Alliance”  “Dementia care”  “Dementia Australia- local office”  “Better Hearing Australia/Soundfair”  “AABH” |
| T1b | “University course and In house training at work”  “Special interest group support network”  “Psychology degree”  “Covered in masters course content and on the job training as part of a research project looking at hearing loss and cognition”  “Cognate coursework” |

**Exploratory Factor Analysis of KAP Survey**

In order to determine how to score the KAP survey, we conducted an exploratory factor analysis (EFA). To foreshadow, we concluded from the EFA results that item-level analysis was more suitable for the KAP-survey data, as the identified factors were difficult to neatly define (i.e., they were not pure knowledge, attitude, and practice factors). Furthermore, the KAP survey was not originally designed to measure underlying knowledge, attitude, and practice factors per se; rather, it was designed to gather information on points of interest related to considerations of cognitive impairment in audiological practice. However, for transparency, and to benefit future research, we have provided the EFA results here. The EFA was run using SPSS 29 and O’Connor’s syntax for parallel analysis (O’connor, 2000). Parallel analysis, with 5000 permutations of the raw dataset, was used to identify the correct number of factors to extract; the criterion used for extraction was a raw-data eigenvalue larger than the upper 95% CI for the equivalent random-data eigenvalue. For extraction, we utilised maximum-likelihood estimation with direct-oblimin rotation and Kaiser normalisation.

The KMO estimate for sampling adequacy (Kaiser & Rice, 1974) and Bartlett’s test of sphericity indicated that our KAP Likert-scale data were suitable for factor analysis (*KMO* = .74; χ^2^ [351] = 1154.079, *p* < .001). Parallel analysis indicated that a five-factor solution was appropriate for our data. As shown in Table S2, communalities ranged from .07 to .84, and the final, rotated factor solution demonstrated an acceptable degree of simple structure. As further shown in Table S1, while factors 4 and 5 were composed solely of practice and attitude items, respectively, not all practice and attitude items were captured by these factors. Indeed, the remaining three factors contained some mixture of knowledge, attitude, and/or practice items. This result was likely due to certain thematic elements being common across question types, namely: (1) awareness of, and access to, cognitive tools; (2) perceived worth of engaging clients in conversations about their cognition, and tendency to do so; (3) awareness of potential cognitive issues in clients; (4) resources available to accommodate cognitive issues in practice; and (5) perceived relevance of cognitive issues in audiology. As shown in Table S3, the extracted factors mostly correlated positively, with only one negative correlation between factors 4 and 5; though, several correlations were likely non-significant, as their effect sizes were lower than .10. In sum, it is perhaps unsurprising that knowledge, attitude, and practice (i.e., behaviour) factors were not found, as these attributes are highly interrelated and thus harder to tease apart in measurement. However, with more-deliberate construction of questions, future research may be able to create an audiological KAP survey that produces knowledge, attitude, and, practice scores.

**Table S2.** *Extracted communalities and fully standardised factor loadings for the KAP survey data.*

|  | | Factor 1 | Factor 2 | Factor 3 | Factor 4 | Factor 5 |
| --- | --- | --- | --- | --- | --- | --- |
| **Question** | *h*^2^ | λ | λ | λ | λ | λ |
| K9 | .46 | **.67** | .01 | -.04 | .05 | -.03 |
| K8 | .63 | **.63** | .35* | -.01 | -.11 | -.04 |
| K5 | .56 | **.60** | -.04 | -.05 | -.22* | .33* |
| P7 | .51 | **.59** | -.11 | .18 | .36* | -.14 |
| K7 | .43 | **.56** | .03 | -.04 | .20* | .15 |
| K11 | .31 | **.46** | -.06 | -.06 | .30* | .10 |
| P4 | .48 | **.42** | .34* | -.25* | -.14 | .19 |
| K2 | .26 | **.36** | .12 | .29* | -.10 | -.03 |
| K4 | .20 | **.29** | .09 | .13 | -.20* | .15 |
| A2 | .84 | .03 | **.91** | .15 | -.11 | -.11 |
| A2a | .78 | .24* | **.82** | .01 | -.10 | -.11 |
| P2 | .72 | -.06 | **.66** | .03 | .34* | .19 |
| A1 | .65 | -.28* | **.63** | .13 | .13 | .36* |
| P6 | .51 | -.02 | **.52** | -.10 | .37* | .14 |
| P3 | .45 | .38* | **.42** | -.01 | .08 | .06 |
| K3 | .67 | -.04 | -.13 | **.78** | -.13 | .19 |
| K6 | .55 | .02 | -.06 | **.66** | .02 | .27* |
| K1 | .35 | .08 | .17 | **.55** | -.03 | -.19 |
| K10 | .31 | -.14 | -.08 | **.40** | .35* | .01 |
| A3 | .16 | -.01 | .11 | **.33** | .12 | .01 |
| P9 | .45 | .19 | .08 | .23* | **.53** | .00 |
| P1 | .41 | .02 | .36* | -.09 | **.44** | .11 |
| P8 | .25 | .01 | .12 | .17 | **.40** | .01 |
| P10 | .07 | .06 | .03 | -.09 | **.22** | -.11 |
| A5 | .67 | .13 | -.05 | .01 | -.01 | **.80** |
| A4 | .56 | -.05 | .07 | .04 | .02 | **.73** |
| A6 | .53 | .11 | .04 | .07 | -.04 | **.68** |

*Note*: *, cross-loadings ≥ .20; Bolded loadings represent the most-significant loadings for each factor.

**Table S3.** *Inter-factor correlations.*

|  | Factor 1 | Factor 2 | Factor 3 | Factor 4 | Factor 5 |
| --- | --- | --- | --- | --- | --- |
| Factor 1 | - |  |  |  |  |
| Factor 2 | .30 | - |  |  |  |
| Factor 3 | .05 | .13 | - |  |  |
| Factor 4 | .06 | .20 | .10 | - |  |
| Factor5 | .17 | .22 | .14 | -.04 | - |

*Note*: SPSS does not provide statistical significance for factor correlations.

**Refrences**

Kaiser, H. F., & Rice, J. (1974). Little Jiffy, Mark Iv. *Educational and Psychological Measurement*, *34*(1), 111-117. https://doi.org/10.1177/001316447403400115

O’connor, B. P. (2000). SPSS and SAS programs for determining the number of components using parallel analysis and Velicer’s MAP test. *Behavior Research Methods, Instruments, & Computers*, *32*(3), 396-402. https://doi.org/10.3758/BF03200807
